# FARSITE: evaluation of an automated trial feasibility assessment and recruitment tool

**DOI:** 10.1186/1745-6215-12-S1-A113

**Published:** 2011-12-13

**Authors:** Sarah Thew, Gary Leeming, John Ainsworth, Martin Gibson, Iain Buchan

**Affiliations:** 1 North-west e-Health, Manchester, UK; 2 Community Based Medicine, University of Manchester, Manchester, UK; 3 Greater Manchester Comprehensive Research Network, Manchester, UK

## Objectives

In a review of UK-supported clinical trials more than half of the investigators asked the funding agency for an extension and a third did not hit their recruitment targets [1]. Study feasibility is often assessed on an ad hoc basis by asking clinical staff to estimate how many patients with particular characteristics they might expect to see in a given time period, and over-estimation is common. We have developed FARSITE (Feasibility Assessment and Recruitment System for Improving Trial Efficiency), a system to support the evaluation of trial feasibility by providing accurate assessments of numbers of patients eligible for a particular trial. Furthermore FARSITE automates patient recruitment whilst preserving consent for consent.

## Methods

Previously we investigated the requirements of clinical and protocol development experts for a system to support trial design and recruitment. We developed a software architecture which addresses their primary concerns: preservation of consent for consent; improving the efficiency of the trial design process and automation of as much as possible of the recruitment workflow [2]. The FARSITE software is based on this architecture and has been deployed in collaboration in the Greater Manchester Clinical Research Network and the NHS in Salford. We present the results of an analysis of the benefits of using the FARSITE application when compared to previous modes of working.

## Results

We have worked with local clinical research organisations to evaluate FARSITE, running recruitment criteria for on-going clinical trials through FARSITE and comparing our estimates of numbers of patients eligible for the trial with the trials’ actual recruitment rates. A strong correlation was observed between protocols with a low FARSITE recruitment estimate and trials struggling to recruit participants

## Conclusions

We have shown that FARSITE can improve the speed and efficiency of clinical trials feasibility, allowing researchers to quickly assess the feasibility of trials in advance.
